# Interactive effects of rising temperatures and urbanisation on birds across different climate zones: A mechanistic perspective

**DOI:** 10.1111/gcb.16645

**Published:** 2023-03-13

**Authors:** Petra Sumasgutner, Susan J. Cunningham, Arne Hegemann, Arjun Amar, Hannah Watson, Johan F. Nilsson, Martin N. Andersson, Caroline Isaksson

**Affiliations:** ^1^ Konrad Lorenz Research Centre, Core Facility for Behavior and Cognition, Department of Behavioral & Cognitive Biology University of Vienna Vienna Austria; ^2^ FitzPatrick Institute of African Ornithology, DSI‐NRF Centre of Excellence University of Cape Town Cape Town South Africa; ^3^ Department of Biology Lund University Lund Sweden

**Keywords:** Anthropocene, anthropogenic resources, behavioural plasticity, human‐induced rapid environmental change (HIREC), immune system, pollution, redox system, urban heat island effect (UHI)

## Abstract

Climate change and urbanisation are among the most pervasive and rapidly growing threats to biodiversity worldwide. However, their impacts are usually considered in isolation, and interactions are rarely examined. Predicting species' responses to the combined effects of climate change and urbanisation, therefore, represents a pressing challenge in global change biology. Birds are important model taxa for exploring the impacts of both climate change and urbanisation, and their behaviour and physiology have been well studied in urban and non‐urban systems. This understanding should allow interactive effects of rising temperatures and urbanisation to be inferred, yet considerations of these interactions are almost entirely lacking from empirical research. Here, we synthesise our current understanding of the potential mechanisms that could affect how species respond to the combined effects of rising temperatures and urbanisation, with a focus on avian taxa. We discuss potential interactive effects to motivate future in‐depth research on this critically important, yet overlooked, aspect of global change biology. Increased temperatures are a pronounced consequence of both urbanisation (through the urban heat island effect) and climate change. The biological impact of this warming in urban and non‐urban systems will likely differ in magnitude and direction when interacting with other factors that typically vary between these habitats, such as resource availability (e.g. water, food and microsites) and pollution levels. Furthermore, the nature of such interactions may differ for cities situated in different climate types, for example, tropical, arid, temperate, continental and polar. Within this article, we highlight the potential for interactive effects of climate and urban drivers on the mechanistic responses of birds, identify knowledge gaps and propose promising future research avenues. A deeper understanding of the behavioural and physiological mechanisms mediating species' responses to urbanisation and rising temperatures will provide novel insights into ecology and evolution under global change and may help better predict future population responses.

## Graphical abstract

Resource availability/reliability (blue) and quality (purple) differ between urban and non‐urban areas and will interact with rising temperatures to influence the magnitude of biological impacts of climate change. Specifically, the urban environment could both buffer and exacerbate the biological impacts of rising temperatures. Greater water availability/reliability in cities buffers thermoregulatory costs, while poorer urban water quality exacerbates warming‐related increases in disease risk and pollutants may reduce thermoregulatory capacity. Greater food availability and artificial light at night may buffer foraging‐thermoregulation trade‐offs in cities, while warming‐related declines in food quality (e.g. carotenoids) are exacerbated in cities. Urban nest‐ and roost sites are poorly thermally buffered, hence of lower quality, than non‐urban sites, while more shade is available in cities, especially in arid regions. Finally, the effects of rising temperatures are likely exacerbated by air pollution and vice versa, with impacts on the redox system and immune system. The UHI effect under climate change will likely continue to lead to relaxed thermoregulatory costs in temperate, continental and polar cities during winter, while exacerbating thermoregulatory costs in summer and in tropical cities compared with non‐urban environments. Cities in arid zones often have a reversed heat island effect, buffering thermal costs.

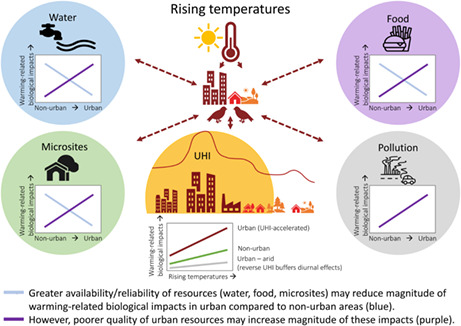

## 1 INTRODUCTION

Climate change and urbanisation represent two of the greatest ongoing challenges for organisms (United Nations, 2019). However, they are typically considered in isolation, with little empirical research on how their interaction may influence behaviour, physiology and ultimately fitness (but see Becker & McCluney, 2021; Diamond et al., 2014; Stofberg et al., 2022). The critical importance of this knowledge gap was recently highlighted in a horizon‐scanning exercise, which identified climate–urban interactions as one of six core emerging themes in urban evolutionary ecology (Verrelli et al., 2022). Cities might be an appropriate system to develop and test hypotheses about the biological effects of climate change (Diamond et al., 2017; Diamond & Martin, 2021; Rivkin et al., 2019; Youngsteadt et al., 2015) given the ***urban heat island*** ***effect*** (hereafter ‘***UHI***’; Oke, 1982). However, increases in temperature associated with climate change could have different effects on organisms in ***urban*** versus ***non‐urban*** environments. For example, in urban settings, the effects of elevated temperatures could be buffered by greater availability or higher reliability of resources such as water, food or microsites (i.e. locations that differ in their microclimate on a small spatial scale), which may be limited or distributed differently in non‐urban environments. Alternatively, the impacts of warming could be exacerbated by urban‐related factors such as lower quality of water, food or microsites, higher levels of pollutants and the UHI.

The direction and magnitude of impacts on wildlife of globally elevated temperatures under climate change likely vary across cities located in different ***climate types***. For example, elevated temperatures could result in positive fitness outcomes for urban animals in one region, but negative effects in another region, depending on baseline climate conditions. In temperate, continental and polar regions, the combination of the UHI and increasing temperatures could offer more favourable thermal environments, especially during winter, compared with non‐urban areas. In contrast, in tropical or arid regions, elevated temperatures could push animals beyond thermal thresholds, above which immediate survival and long‐term fitness are compromised (Mitchell et al., 2018), especially within the UHI. Regional differences might also arise due to underlying ecological and evolutionary differences between taxa in the Northern *versus* Southern Hemisphere (e.g. differences in plumage pigmentation, breeding systems, female song and aggression, etc., Theuerkauf et al., 2022). While we recognise that climate change is more than ***anthropogenic global warming*** and also influences other climatic factors besides temperature (e.g. precipitation, wind, ocean currents, etc.), those changes are far less predictable in time and space compared with rising temperatures, which makes it difficult to create coherent predictions. Thus, for this review, we focus on the more well‐known impacts of temperature, while acknowledging that other climate variables will also interact with the urban environment.

Relatively few empirical studies explore the biotic effects of climate change simultaneously in urban and non‐urban habitats (but see Diamond et al., 2014; Zohner, 2019 on phenology; Oliver & Morecroft, 2014 on biodiversity; and Pipoly et al., 2022 on reproduction). Furthermore, none have considered how climate change impacts may differ between cities in different climate types and/or geographical regions. This is surprising, given that differences in organismal thermal physiology that could affect climate change responses between climate types have been documented: for example, lower metabolic rates in tropical, compared with temperate, bird species (Wiersma et al., 2007). Finally, temperature extremes, which are predicted to increase in frequency, duration and amplitude with climate change (Drumond et al., 2020; Perkins‐Kirkpatrick & Lewis, 2020; Rahmstorf & Coumou, 2011; Ummenhofer & Meehl, 2017), are potentially more important for abrupt changes in ecological systems than mean temperature increases over time (Turner et al., 2020) and can be further accelerated by the UHI (Depietri et al., 2011). Such temperature extremes may be the greatest drivers of selection on physiological traits with corresponding fitness consequences for animals (Stager et al., 2016; Vasseur et al., 2014; Wingfield et al., 2017).

Here, we first briefly summarise current knowledge of the separate effects of elevated temperatures and urbanisation on bird behaviour and physiology. We focus on individual‐level mechanistic responses that can mediate changes in fitness, rather than population‐ and community‐level responses, although we recognise that these other processes also operate. We then address the impact of the UHI across different climate types, and finally, we explore how the effects of elevated temperatures may differ between urban and non‐urban areas and make predictions for the interactive effects of elevated temperatures and urbanisation.

We focus on birds throughout, as they are an excellent taxonomic group to unravel the impacts of urbanisation (Lepczyk et al., 2017) and elevated temperatures (Møller et al., 2010)—and will, therefore, be well suited to explore their potential interactive effect. Birds are prominent in both urban and non‐urban faunal communities (Aronson et al., 2014), easily observable and express a diverse range of responses to changes in urban ecosystems (Aronson et al., 2014; Gil & Brumm, 2014; Isaksson & Bonier, 2020; Marzluff, 2001) and temperature (McKechnie, Gerson, et al., 2021; Pollock et al., 2021; Sauve et al., 2021). Consequently, they can serve as an ideal model taxon that may provide insight into the responses of other taxa. However, in some cases, examples from avian taxa are rare or non‐existent in the literature, especially regarding the interactive effects of pollutants and temperature on animal physiology. In these cases, we draw from the literature on other taxa. Our overall aim is to stimulate further empirical research in this understudied, yet increasingly important, area of global change biology. Throughout, we highlight terms in ***bold and italic*** where more detailed definitions can be found in the supplementary glossary.

### 1.1 Behavioural and physiological responses to elevated temperatures

**FIGURE 1:**
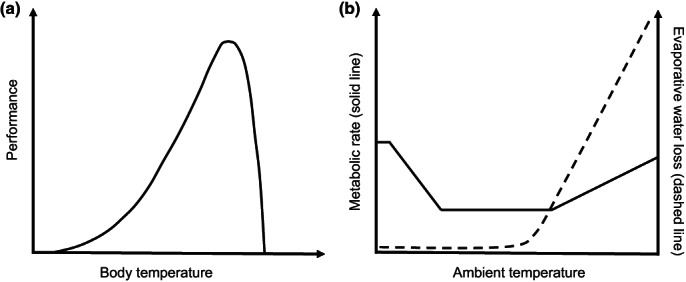
(a) ***Thermal performance curve***: Physiological performance is dependent on body temperature in a strongly non‐linear way. All animals have a range of ‘optimum’ body temperatures across which physiological performance is maximised. Performance declines at body temperatures outside of this range, and loss of function is more precipitous at hyperthermic than hypothermic body temperatures. (b) ***Scholander–Irving model*** of endotherm thermoregulation: Metabolic and evaporative water loss rates change strongly non‐linearly with increasing ambient temperature, allowing endotherms to maintain stable body temperatures across a range of ambient temperatures by increasing or decreasing metabolic heat production and evaporative heat loss.

Temperature is a fundamental driver of biological processes (Sexton et al., 2009). Biological responses to temperature variation are strongly non‐linear and often quadratic (Figure 1) and include performance‐related responses to body temperature (Figure 1a) and behavioural and physiological thermoregulatory responses to environmental temperatures (Figure 1b). Because of these non‐linear relationships, increased temperatures in cold environments can relax thermoregulatory costs allowing energy to be allocated to other fitness‐enhancing processes, such as the ***immune system***, resulting in improved fitness outcomes. In contrast, negative effects can occur in environments that are already hot (e.g. Catry et al., 2015; Redpath et al., 2002). Thus, the strength and sign (positive or negative) of the impact of a similar‐magnitude increase in ***environmental temperature*** on animals will likely depend on the baseline climate conditions.

In cold environments, birds frequently rely on facultative hypothermia to conserve energy at night (McKechnie & Lovegrove, 2002). Warmer environmental temperatures could allow birds to enter shallower hypothermia (Nord et al., 2011), with positive outcomes such as reduced predation risk (Andreasson et al., 2019; Carr & Lima, 2013), increased immune function (Sköld‐Chiriac et al., 2015) and deeper sleep (Mueller et al., 2012). As cold adaptation is linked to metabolic rate in temperate birds, warmer environmental temperatures may also result in directional selection for reduced basal metabolic rate, which could have cascading effects on other physiological and behavioural traits (Nilsson & Nilsson, 2016). While higher environmental temperatures can have positive effects in advancing egg‐laying dates and shortening incubation periods in some temperate species (e.g. Bleu et al., 2017), other studies suggest many temperate birds are already limited by high environmental temperatures (Mueller et al., 2019; Nord & Nilsson, 2019). Overall, it is likely that the directional impact of rising temperatures in temperate regions might differ between seasons, with largely positive effects in winter and potentially negative effects during summer.

In already hot environments, such as tropical and arid zones where ***air temperatures*** exceeding 30°C are common, extreme heat events can lead to mass mortalities (McKechnie, Rushworth, et al., 2021). Even moderate temperature increases can cause birds to retreat into thermal refuges (e.g. shaded locations Carroll et al., 2015; Kearney et al., 2011; Martin et al., 2015) and make trade‐offs between thermoregulation and other important behaviours (e.g. foraging Cunningham et al., 2021). Negative consequences include reduced cognitive performance (Danner et al., 2021), body mass (du Plessis et al., 2012; van de Ven et al., 2019), nest provisioning rates (Cunningham et al., 2013; Edwards et al., 2015; Wiley & Ridley, 2016), nestling growth (Andrew et al., 2017; Rodríguez & Barba, 2016) and quality and survival of offspring (Cunningham et al., 2013; Wiley & Ridley, 2016) and adults (Bourne et al., 2020). In some systems, these behaviourally mediated costs may be as, or more, important than physiological temperature tolerance limits in determining population persistence under climate change (Conradie et al., 2019). Mass loss associated with foraging‐thermoregulation trade‐offs may also have negative impacts on other physiological systems that are important for homeostasis and self‐maintenance. For example, food‐limited birds can experience weaker constitutive immune function and immune responses (Buehler et al., 2009; Cornelius Ruhs et al., 2019; Eikenaar et al., 2020) and energy‐based trade‐offs between immune function and thermoregulation (Nord et al., 2020).

### 1.2 Behavioural and physiological responses to urbanisation

Urbanisation has been directly linked to several major components of global change, including climate and land‐use change, biological invasions, biodiversity loss and alterations in biogeochemical cycles (Grimm et al., 2008). Urbanisation changes the availability and quality of resources for birds and creates light, sound and chemical pollution (Isaksson, 2018; Luniak, 2004), which can affect organisms directly via physiological constraints and indirectly by disrupting sensory systems (Dominoni et al., 2020) and trophic interactions (Post et al., 2008). Artificial light at night (ALAN) can provide an additional source of heat and has—together with avoidance of human presence—caused a worldwide increase in nocturnality of previously diurnal or crepuscular wildlife (Gaynor et al., 2018).

Although some avian species thrive in urban environments, urban‐dwelling individuals may still face fitness‐related costs of city life: within species, urban individuals show, for example, behavioural differences such as extended daily activity and disrupted sleep due to ALAN (Aulsebrook et al., 2020; Dominoni et al., 2014), higher song amplitude and reduced song repertoire due to sound pollution (Derryberry et al., 2020; Slabbekoorn, 2013; Slabbekoorn & Ripmeester, 2008) and increased boldness (Atwell et al., 2012; Kumar et al., 2018). Exposure to urban stressors can cause oxidative and genotoxic damage, impairing cellular function and triggering premature senescence (Isaksson, 2015; Watson et al., 2015; also see Sumasgutner et al., 2019). In addition, nutritional physiology has changed in many urban passerines, altering their morphology (e.g. smaller body sizes Caizergues et al., 2021; shorter or narrower beaks De et al., 2014 and shorter wing and tail length Caizergues et al., 2021; Evans et al., 2009a). Furthermore, there is some evidence that thermal physiology has changed in cities, for example, fewer feathers in great tits (*Parus major*) (Sándor et al., 2021) in response to the UHI. Urbanisation may reduce dietary availability of carotenoids, which are important for coloration and sexual signalling, development, disease resistance and night vision (Giraudeau et al., 2015; Isaksson et al., 2005; Penteriani & Delgado, 2017).

To date, most research has focused on cities in temperate or continental regions of the Northern Hemisphere. In contrast, data are lacking for cities in tropical and arid regions (Aronson et al., 2016) and for cities in developing countries with different ecologically relevant social gradients (e.g. wealth, socioeconomic gradients or human socio‐cultural factors; Kumar, Gupta, et al., 2019; Reynolds et al., 2021; Sumasgutner, 2021). This reflects the well‐recognised Northern Hemisphere bias in ecological and evolutionary research (Awoyemi & Ibáñez‐Álamo, 2023; Marzluff, 2017; McHale et al., 2013; Shackleton et al., 2021; Theuerkauf et al., 2022).

## 2 RISING TEMPERATURES AND THE URBAN HEAT ISLAND EFFECT

The UHI is the most globally consistent difference in the abiotic environment between urban and adjacent non‐urban areas, causing increased ***air and environmental *temperatures**** and reduced diel temperature fluctuations in urban environments (reviewed by Arnfield, 2003; Climate Central, 2021; Grimmond, 2007; Heisler & Brazel, 2015; Imhoff et al., 2010; Martilli et al., 2020). For instance, cities in the United States can be up to 8°C warmer than the surrounding countryside, especially in summer (air temperature on average 4.3°C warmer, compared with 1.3°C in winter; Imhoff et al., 2010). This UHI effect might drive directional selection for greater physiological thermal tolerance, which could ultimately genetically pre‐adapt urban populations to a warmer future (Johnson & Munshi‐South, 2017; Lambert et al., 2021).

### 2.1 Temperate, continental and polar cities and relaxed thermal constraints

In temperate, continental and polar cities, the UHI may relax thermoregulatory costs and could, thus, facilitate avian settlement, buffer against cold winters and extend the breeding season (Chace & Walsh, 2006; Shochat et al., 2006). Relaxation of thermoregulatory costs due to the UHI and climate warming, and/or supplementary feeding during winter, may also reduce the propensity of urban birds to migrate. For example, urban blackbirds (*Turdus merula*) have shorter wings than non‐urban blackbirds in Europe, perhaps due to their increased residency (Evans et al., 2009a). Migratory strategies are independently changing towards residency in the face of a global temperature increase, as demonstrated for European blackcaps (*Sylvia atricapilla*) (Plummer et al., 2015). However, in urban areas, it is not yet known if the main driver for these changes in migratory behaviour is temperature or food abundance, that is, supplementary feeding during winter (Evans et al., 2009a; Partecke & Gwinner, 2007; see also Section 3.2). In either case, changes in migratory propensity and increased residency are likely to be linked to a suite of behavioural, physiological and morphological adaptations that can alter fitness aspects (reviewed by Goossens et al., 2020; Hegemann et al., 2019; Piersma & Van Gils, 2011).

Furthermore, milder winters are associated with changes in predator‐prey or host–parasite interactions (Williams et al., 2015), and those effects might be particularly accelerated in urban habitats due to the UHI. For example, rural tawny owls (*Strix aluco*) in Finland bred at lower frequency after colder winters, while urban conspecifics were less influenced by local weather conditions, apparently buffered by the UHI and wider prey availability in the city (Solonen & af Ursin, 2008). Likewise, milder winters could change pathogen assemblages and host–parasite interactions, especially in urban areas (Altizer et al., 2013; Hall, 2021; Kutz et al., 2009), by altering vector abundance or emergence in a warming world (Harvell et al., 2002; Medlock & Leach, 2015). This could be particularly problematic for species that are adapted to low pathogen diversity and/or transmission, as, for instance, those living at higher latitudes and hence selected for low immune function (Nord et al., 2020).

Finally, temperature changes may cause shifts in avian phenology. This pattern occurs not only spatially along urban to non‐urban gradients (Chamberlain et al., 2009) but also temporally: as spring temperatures have increased in the Northern Hemisphere in recent decades, phenology has advanced due to relaxation of thermal constraints and associated changes in prey availability (Bates et al., 2023; Parmesan, 2007; Thackeray et al., 2016).

### 2.2 Tropical and arid cities and increased thermal stress

The frequency, duration and amplitude of heat waves are increasing with climate change (Meehl & Tebaldi, 2004; Schär et al., 2004) with heat‐related mass mortalities occurring in both urban (Kassam, 2022) and non‐urban systems (McKechnie, Rushworth, et al., 2021). Such events are projected to occur more frequently in the future (Conradie et al., 2020) and might be more severe in already hotter urban centres. Interventions intended to reduce heat stress for humans in urban environments could also aid urban wildlife. For example, amelioration of the UHI in summer can be aided by the provision of trees for shade (Pena et al., 2017), sprinkling streets to increase evaporative cooling, increasing the albedo effect by employing light‐coloured paint on roofs, the replacement of open parking lots with garages (Pacione, 2001) and green or blue‐green roofs (i.e. with an extra blue water retention layer underneath the green layer; Busker et al., 2022). The utility of vegetation to improve urban climates is widely acknowledged (Aronson et al., 2014), and temperatures under trees can be up to 40°C cooler than on non‐shaded asphalt surfaces (Rahman et al., 2020). This, together with an abundance of artificial water resources in urban areas (see Section 3.1), can lead to relaxation of heat stress for urban wildlife.

Cities in arid and semi‐arid regions show a modified UHI effect, often exhibiting lower temperatures than surrounding non‐urban areas during the day (‘***urban heat sink***’ Carnahan & Larson, 1990; Imhoff et al., 2010; Nassar et al., 2016) but with a pronounced heat island effect at night. In semi‐arid and arid locations, we expect cities to become important thermal refugia as the climate warms, therefore exposing more individuals to alternative risks associated with anthropogenic infrastructure and urban pollution (see below). Some neotropical migrants, including species of conservation concern, already use urban centres as non‐breeding habitats and exploit urban parks and vegetation structures within cities (MacGregor‐Fors et al., 2010). While this might so far be mainly driven by changes in land‐use, the urban heat‐sink effect in hot regions could potentially increase the pressure on species to move into cities with rising temperatures.

## 3 THE INTERACTIVE EFFECT OF TEMPERATURE AND URBANISATION

**FIGURE 2:**
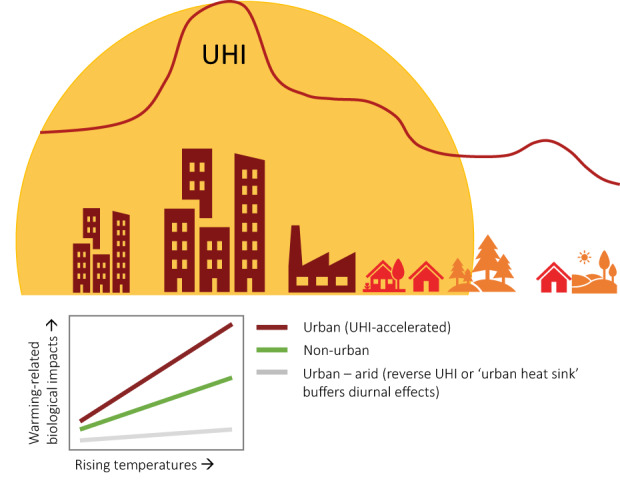
Predicted interactive effects between rising temperatures and urbanisation in relation to the urban heat island (UHI) effect: Birds in urban areas might be genetically pre‐adapted to a warmer future through experiencing selective pressures of the UHI. The UHI might also result in accelerated temperature‐related changes to migration, phenology and pathogen assemblages, and an earlier onset of deleterious heat stress including heat‐related mortality. Cities in arid and semi‐arid regions show a modified UHI effect, often exhibiting lower temperatures than non‐urban areas during the day, buffering the above‐mentioned diurnal effects. The UHI under globally warming temperatures will likely continue to lead to relaxed thermoregulatory costs in temperate, continental and polar cities during winter, while exacerbating thermoregulatory costs in summer and in tropical cities compared with non‐urban environments.

**FIGURE 3:**
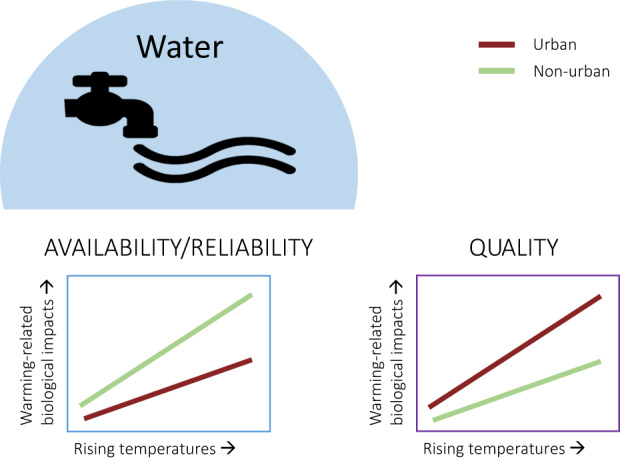
Predicted interactive effects between rising temperatures and urbanisation in relation to water resources: Greater surface water availability and reliability in cities buffers thermoregulatory costs, which could result in reduced biological impacts of warming in cities versus non‐urban areas (left). However, a poorer urban water quality exacerbates warming‐related increases in disease risk, and exposure to pollutants in urban water sources might reduce thermoregulatory capacity, therefore, increasing the costs of heat exposure in urban versus non‐urban areas (right).

**FIGURE 4:**
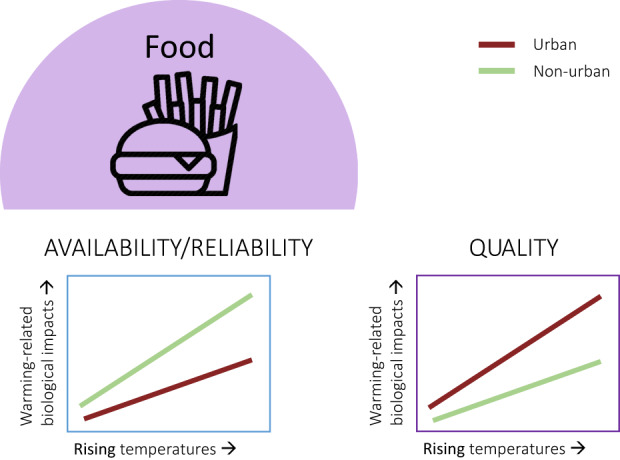
Predicted interactive effects between rising temperatures and urbanisation in relation to food resources: Greater food availability may buffer foraging‐thermoregulation trade‐offs by improving foraging efficiency and artificial light at night in cities may allow foraging during cooler times of day (e.g. extending ‘daylight’ before dawn and after dusk) for diurnal birds, reducing biological impacts of warming temperatures in urban versus non‐urban areas (left). However, high temperatures may reduce food quality (e.g. trees synthesise lower carotenoid content under heat reducing carotenoid availability across the food web), which can be exacerbated in cities by the urban heat island effect and air pollution, causing increased biological impact of warming in urban than non‐urban areas (right).

**FIGURE 5:**
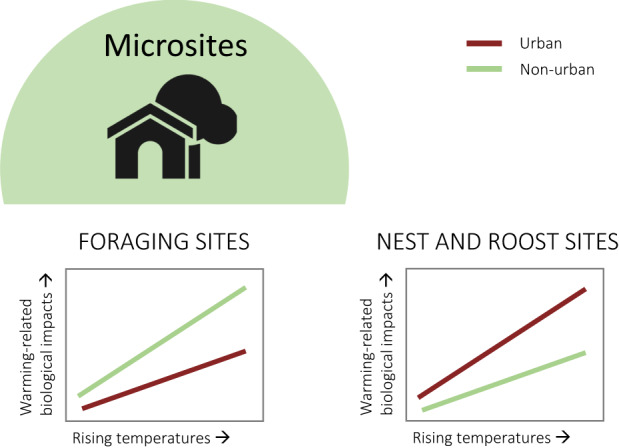
Predicted interactive effects between rising temperatures and urbanisation in relation to microsite availability and quality: Greater shade availability in cities, especially in arid regions, may buffer foraging‐thermoregulation trade‐offs especially for diurnal animals and reduce the thermal costs associated with rising temperatures in urban versus non‐urban areas (left). However, urban nest‐ and roost sites are poorly thermally‐buffered making them of lower quality compared with natural sites with implications for individual health and fitness outcomes and potentially exacerbating warming‐related biological impacts in urban areas versus non‐urban areas (right).

**FIGURE 6:**
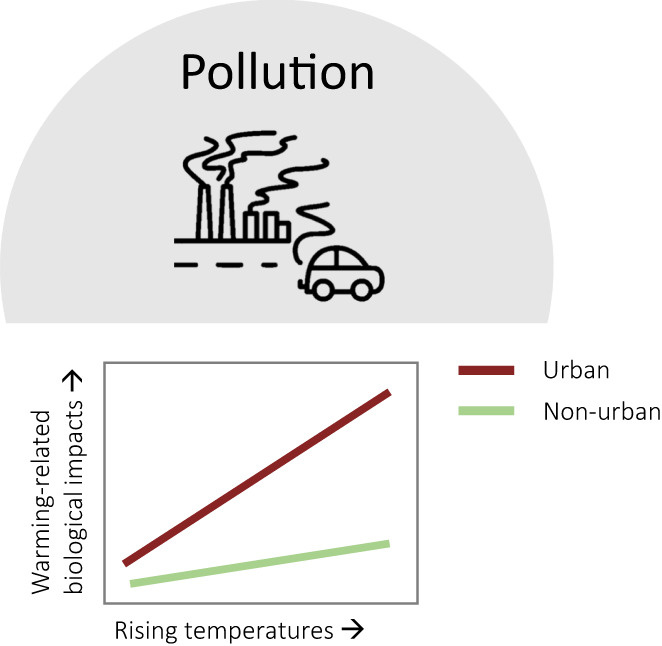
Predicted interactive effects between rising temperatures and urbanisation in relation to air pollution: The redox system, important in mitigating the negative health effects of air pollution, is in itself negatively affected by rising temperatures, making the impact of global temperature rise in urban areas more severe than in non‐urban areas. In addition, high temperatures can compromise immune function, which is already challenged by air pollution and exposure to modified pathogen assemblages in urban versus non‐urban environments.

In the following sections, we discuss the important environmental differences between urban and non‐urban systems in the context of globally rising temperatures. Resource availability and quality, in terms of water, food and microsites, and other abiotic factors like air pollution—all altered in urban areas—may strongly interact with rising temperatures to affect bird behaviour and physiology, especially as the UHI further elevates temperatures within urban areas (Figure 2). In Section 3.1, we contrast higher surface water availability/reliability in urban areas—due to higher precipitation and abundant artificial water resources, with lower water quality—due to higher nutrient, heavy metal and pollutant loads, lower turbidity and reduced aquatic biodiversity (McKinney, 2002; Roy et al., 2003; Sullivan et al., 2021) compared with non‐urban areas. All these factors interact and may be exacerbated by increasingly higher water temperatures (Figure 3). In Section 3.2, we contrast the abundance of anthropogenic food sources (Risi et al., 2021) with the quality of these resources in urban areas. Anthropogenic food can sometimes compensate partly or wholly for an overall scarcity of natural foods (Plummer et al., 2019; Seress et al., 2020) but may be of lower quality due to a lack of essential micronutrients in processed food (Coogan et al., 2018), which can lead to impaired health (Murray et al., 2016). Interactive effects between urban food resources and temperature may become apparent through behavioural trade‐offs between foraging, heat dissipation and water loss (Figure 4). In Section 3.3, we shed light on small‐scale variations in the microclimate between locations that birds use for foraging, self‐maintenance like preening or roosting, and breeding, between urban and non‐urban sites. These sites can have different thermal properties and ultimately influence the heat load birds will experience in a warming world (Figure 5). Finally, we explore the interaction of air pollution (higher in urban environments, Section 3.4) and rising temperatures on the immune and ***redox systems***, predicting exacerbated impacts on birds' health in urban centres due to these effects (Figure 6).

### 3.1 Water resources

#### 3.1.1 Water availability

Rainfall is approximately 5%–10% higher in cities than in surrounding non‐urban areas because of greater air turbulence and a higher concentration of dust particles (the urban dust dome) that serve as hygroscopic nuclei (Pacione, 2001). Moreover, urban areas often contain parks and gardens with artificial watering (or drought‐proofing), providing a reliable water resource, which increases and stabilises primary productivity throughout the system (Imhoff et al., 2000) and could create valuable refugia for wildlife during droughts—especially in arid regions and as temperatures continue to rise. A recent review exploring the pattern of higher biodiversity in urban areas with wealthier citizens found that the ‘***luxury effect***’ was most pronounced in arid zones, suggesting that the availability of water in wealthy neighbourhoods could be the driving mechanism (Chamberlain et al., 2020). Differences in water availability between urban and non‐urban areas may become particularly important as global temperatures rise because evaporative water loss plays an important role in thermoregulation. It is the sole mechanism for birds to dissipate heat when environmental temperatures exceed body temperature (McKechnie et al., 2012; McKechnie & Wolf, 2010). Birds gain water through drinking, from the moisture contained in food (preformed water) or from water released during metabolism (metabolic water). Reliance on surface water for drinking increases with air temperature, and some species shift from obtaining all water from food to drinking, when open water sources are available (Pattinson et al., 2020; Smit et al., 2013). Within‐species, hydrated individuals may maintain lower body temperatures (Maloney & Dawson, 1998). Thus, in urban environments, increased access to reliable water may facilitate birds' ability to maintain stable body temperatures in hot weather, increasing their capacity to remain active and reducing their exposure to foraging‐thermoregulation trade‐offs and buffering climate warming impacts compared with non‐urban environments. Such benefits would likely be greater in already‐hot tropical and arid cities and less important in temperate, continental and polar cities. The importance of water resource availability in arid regions is starkly illustrated by a study of Mojave Desert bird biodiversity, in which the presence of water partially buffered avian communities from climate‐change‐driven collapse over the last century (Iknayan & Beissinger, 2018). A similar mechanism could be expected to help preserve bird communities in urban areas.

#### 3.1.2 Water quality

Water quality may differ between urban and non‐urban areas in terms of both pathogen abundance and diversity (Delgado‐V & French, 2012; Evans et al., 2009b) and pollutants. Pathogens can accumulate in urban wastewater and local water bodies, and this may be exacerbated by higher temperatures (e.g. due to acceleration of pathogen development times) and, thus, could have significant consequences under climate warming. Indeed, rising temperatures are already associated with an increase in infectious diseases in many ecosystems (e.g. Altizer et al., 2013; Harvell et al., 2009). For example, relevant disease vectors for human health, such as malaria‐spreading *Anopheles* mosquitoes breeding in standing water or filariasis‐spreading *Culex* mosquitoes breeding in blocked drains, latrines or septic tanks both profit from higher temperatures in urban areas (McGranahan et al., 2001). This, together with inadequate sanitation, still common in cities in developing countries, can lead to high contamination rates (Pacione, 2001).

Transmission of disease can also be higher in urban, compared with non‐urban, areas due to higher host densities, higher concentrations of hosts at feeding and drinking sites, and increased interactions between wildlife, livestock, pets and humans (Bradley & Altizer, 2007; Franklin et al., 2021). Because pathogens and parasites also react to climate warming, infected birds may have to invest more resources in self‐maintenance (behaviourally and physiologically via immune defences) to counteract parasite loads, which could potentially impair their ability to effectively thermoregulate. Some empirical evidence suggesting interactive effects of climate warming and anthropogenic change may exacerbate disease risk in birds comes from a study on land‐use change (transformation from forest to farmland), climate warming (modelled 2°C temperature increase) and biological invasions of mosquito vectors on Hawaii, which found increased avian malaria transmission resulting in anthropogenic extinctions (Benning et al., 2002). To date, we lack comprehensive literature on the potentially synergistic effect of urbanisation and warming temperatures on physiological and behavioural adaptations to limit the negative effect of increased pathogen exposure. Studies comparing physiological responses to natural and experimental pathogen exposure of urban and non‐urban birds exposed to different temperatures might shed light on this.

Urban water sources may also carry higher pollutant loads, including lead (Pb; Brown et al., 2016; Levin et al., 2021), than those in non‐urban areas, with the potential to affect behaviour and physiology of birds in ways that may be exacerbated by rising air temperatures. Urban air pollution—which correlates with weather and climate variability through deposition or ventilation, regional transport and atmospheric chemistry (Seo et al., 2018)—exacerbates the problem of accumulating heavy metals in water, as atmospheric deposition adds to already polluted stormwater runoffs (Liu et al., 2018). Exposure to heavy metals and other toxins may reduce the capacity of animals to tolerate high body temperatures (evidence from laboratory mammals; Gordon et al., 1987, 1988), likely making the risks of hot environmental temperatures more acute (Dearing, 2013). This mechanistic link has yet to be fully investigated in free‐ranging birds, but it could exacerbate the negative impacts of higher temperatures associated with climate warming in urban settings where birds are likely to carry higher levels of heavy metals in their bodies (Bauerová et al., 2020; Brahmia et al., 2013; Scheifler et al., 2006; Sriram et al., 2022; Sullivan et al., 2021). Birds are affected by toxin ingestion through drinking and accumulative effects (e.g. ingestion of contaminated aquatic invertebrates; Manning & Sullivan, 2021). Thus, higher environmental temperatures make the risks of poisoning more acute (Dearing, 2013), especially in urban areas. Furthermore, recent work suggests higher pollutant loads may make birds less resilient to pathogens (Teitelbaum et al., 2022), exacerbating the issues outlined in Section 3.1.2 As with our suggestion of future research on pathogens (see above), similar experimental manipulations of pollutants in birds exposed to different climatic conditions could help better understand these potential impacts.

### 3.2 Food resources

#### 3.2.1 Food availability

In urban areas, anthropogenic food sources are often abundant (Risi et al., 2021) and may (partly or wholly) compensate for an overall scarcity of natural foods (Plummer et al., 2019; Seress et al., 2020). The provisioning of anthropogenic food may be deliberate, for example, in the form of bird feeding by the public (Reynolds et al., 2017), which occurs mainly in Northern Europe, North America and many Commonwealth countries (Baverstock et al., 2019); or unintentional, such as the discarding of food or refuse (Auman et al., 2008; Kumar, Singh, et al., 2019; Oro et al., 2013; Stofberg et al., 2019), which occurs globally. Abundant anthropogenic food can also lead to high population densities of a few urban‐exploiting bird species, which, in turn, can profit urban bird‐eating raptors (Kettel et al., 2018; Schütz & Schulze, 2018; Suri et al., 2017). Higher food availability in urban areas may affect the behaviour and physiology of birds in multiple ways, including via impacts on time budgets, mass maintenance and glucocorticoid levels. All these can also be influenced by temperature, suggesting interactions between urbanisation and rising temperatures in the context of food availability are likely.

Birds are known to trade‐off heat dissipation behaviours and foraging (e.g. du Plessis et al., 2012, van de Ven et al., 2019). The abundance of easily accessible, predictable, calorie‐rich anthropogenic food resources (Anderies et al., 2007; Møller, 2009; Shochat et al., 2006) may allow urban birds to spend less time foraging, enabling allocation of more time and energy to cooling behaviours (e.g. shade‐seeking, panting or gular fluttering) and, thus, buffering costs of rising temperatures, especially in already‐hot regions. Evidence that anthropogenic food reduces time spent foraging by urban wildlife, compared with non‐urban conspecifics, comes thus far from studies of black bears (*Ursus americanus*; Beckmann & Berger, 2003 and rhesus macaques *Macaca mulatta*; Jaman & Huffman, 2013). The likely impacts of higher food abundance on birds can be predicted from how supplementary‐fed populations respond to high temperatures, regardless of habitat type. For example, supplementary‐fed hoopoe‐larks (*Alaemon alaudipes*) in the Arabian Desert ceased activity earlier in the day and engaged in thermoregulatory behaviours instead of foraging during hot periods (Tieleman & Williams, 2002). However, buffering effects of anthropogenic food may be less evident during the breeding season, when urban birds might be constrained to finding natural food to raise their young (Catto et al., 2021).

Energetic costs of thermoregulation (and, therefore, food demands) also increase steeply in the cold (Figure 1b). In temperate, continental and polar regions, provision of anthropogenic food is most pronounced during winter via deliberate feeding of seeds, nuts and fatballs for small birds in parks and gardens. The consequently increased ease of meeting daily energetic requirements, combined with the UHI, could reduce thermoregulatory costs associated with cold weather for city‐dwelling *versus* non‐urban passerines (e.g. Nilsson et al., 2020; Nord et al., 2011), with potential implications for survival. However, there is currently little empirical data to demonstrate that winter survival of urban passerines is higher compared with those residing in non‐urban habitats (but see Evans et al., 2009; Horak & Lebreton, 1998). In the face of a warming climate, thermoregulatory costs for small birds in winter will be reduced in both urban and non‐urban environments in temperate and polar regions; thus, winter‐feeding‐related energetic advantages in the urban environment may diminish. Additionally, dense predictable food resources often disproportionately benefit larger and or dominant species, while increasing interference competition (Oro et al., 2013). This could cancel out any potential energy savings. In other scenarios, abundant anthropogenic food can reduce intraspecific competition (Oro et al., 2009), and therefore, the outcomes are expected to be highly variable between different avian communities.

Food availability might also be indirectly increased by (artificially) extended daylight hours. In an urban environment, ALAN could allow diurnal birds to alter or extend foraging times in the face of hotter days. Other diurnal taxa show such flexibility. For example, African wild dogs (*Lycaon pictus*) take advantage of moonlit nights to make up for lost foraging time following extremely hot days (Rabaiotti & Woodroffe, 2019). There is evidence for extended foraging activity into the night by a number of urban bird populations (Byrkjedal et al., 2012; DeCandido & Allen, 2006; Kettel et al., 2016; Lebbin et al., 2007; Rejt, 2004; Spelt et al., 2021). While these observations were not linked to temperature, models suggest that diurnal mammals might be able to compensate for climate change by shifting to nocturnal activity (Levy et al., 2019). However, ALAN may also have negative consequences for behaviour and multiple physiological systems, including hormones, immune function and oxidative stress in birds (de Jong et al., 2016; Dominoni et al., 2013; Moore & Siopes, 2000; Raap et al., 2016; Ziegler et al., 2021). Studies investigating correlations between lost foraging time due to heat during the day, increased night‐time activity and potential physiological changes are currently missing for diurnal birds and would add considerably to our understanding on this topic.

Increased food availability in urban areas could also mean negative effects of extreme temperatures on body condition may be less severe in urban *versus* non‐urban birds. Indeed, wild arid‐zone birds showed declines in body mass associated with foraging‐thermoregulation trade‐offs on hot days (du Plessis et al., 2012; van de Ven et al., 2019), whereas similar changes in behaviour for thermoregulation did not translate to changes in body mass in suburban Australian magpies (Edwards et al., 2015) or urban red‐winged starlings (Stofberg et al., 2022). Thus, urban birds' body condition might be better buffered against the effects of warming temperatures in already‐hot environments. An important remaining question is whether resource availability or physiological costs of high temperature, or both, are the limiting factors controlling body mass maintenance and breeding success in urban and nonurban birds. Targeted supplementary‐feeding experiments are required to tease apart the effects of these drivers but are generally lacking to date. However, one experiment in the hot regions of Phoenix, Arizona, United States, shows giving‐up density (i.e. amount of food left behind in food patches after birds ceased foraging) at urban bird feeders is related to air temperature, suggesting that physiological constraints at high air temperatures may limit birds’ access to available food (Shochat et al., 2004).

Anthropogenic food availability may also mediate relationships between rising temperatures and glucocorticoid exposure in birds. For example, increased and more reliable food abundance in urban areas can reduce glucocorticoid levels (Kitaysky et al., 2001; Schoech et al., 2004). Glucocorticoids are a central mediator of metabolism and responses to environmental stressors in vertebrates, and glucocorticoids could be very important in avian responses to urbanisation and climate‐related temperature increases. A robust glucocorticoid response is adaptive, promoting phenotypic and behavioural changes to increase immediate survival, but chronic stimulation of the hypothalamic–pituitary–adrenal axis (and associated secretion of glucocorticoids) can have adverse effects. Acute extreme high temperatures can increase glucocorticoid levels (e.g. Moagi et al., 2021), while chronic harsh thermal conditions might suppress glucocorticoid responses via habituation (de Bruijn & Romero, 2018). The interaction between food availability, temperature and urbanisation could, thus, be particularly important in shaping glucocorticoid regulation and overall glucocorticoid exposure. Several studies have linked baseline glucocorticoid to fitness‐related traits such as reproductive success (Schoenle et al., 2018; Sorenson et al., 2017), and recent experimental studies demonstrate a key role of glucocorticoid regulation in mediating stress resilience and persistence in rapidly changing environments (Vitousek et al., 2019; Zimmer et al., 2020). However, the dynamics of glucocorticoid regulation are highly context‐dependent (Schoenle et al., 2018; Vitousek et al., 2019), and dysregulation of the hypothalamic–pituitary–adrenal axis can be manifest in both increases and decreases in glucocorticoid exposure (Boonstra, 2013). Perhaps unsurprisingly, relationships between urbanisation and glucocorticoid levels in birds are inconsistent (Iglesias‐Carrasco et al., 2020). In the future, it would be valuable to study these complex processes and how they may affect phenotype and fitness in urban *versus* non‐urban birds in the context of rising temperatures.

#### 3.2.2 Food quality

In addition to being highly abundant, the quality of anthropogenic food available in urban centres may differ from the natural diet of birds. For example, certain micronutrients, which are crucial for many physiological systems like oxidative balance (Cooper‐Mullin & McWilliams, 2016; Isaksson, 2020) and immune function (Catoni et al., 2008; Hegemann, Matson, Flinks, et al., 2013; Klasing, 2004; Nwaogu et al., 2020) might be missing from processed anthropogenic food sources, hence constituting threats to the maintenance of homeostasis. Different quantities and quality of food may result in trade‐offs among different physiological systems (i.e. innate immune function *versus* antioxidant defence; Eikenaar et al., 2018, 2019). Potential consequences of these differing food resources in relation to the impacts of rising temperatures are relatively unknown.

***Fatty acid (FA)*** nutrition and physiology have recently been highlighted in the context of urbanisation and temperature variation, specifically regarding winter feeding in urban and suburban areas. The fatty acid composition of tissues and cells affects many physiological processes such as the immune system, growth, thermoregulation and cell membrane function, hence anthropogenic food sources could alter these functions. In seasonally cold environments, plasma fatty acid composition differs between urban and non‐urban passerines, and the direction of the variation is dependent on species and season (Andersson et al., 2015; Isaksson et al., 2017). This variation is likely attributable to differences in food quality and temperature across the urban—non‐urban landscape, both of which directly affect tissue fatty acid composition (Ben‐Hamo et al., 2011). In laboratory experiments with great tits, interactions between air temperature and diets (unsaturated fatty acid diet *versus* saturated fatty acid diet) on basal metabolic rate, oxidative damage and fatty acid biosynthesis were observed (Andersson et al., 2018). These traits are all known to be important for fitness (Isaksson et al., 2011). Hence, warming is likely to have both direct and indirect effects on avian fatty acid physiology, which may be more pronounced in cities where rising temperatures are superimposed on the UHI and diet quality differs from non‐urban habitats. The resulting effects are likely to be dependent on complex interactions between biotic and abiotic factors that differ between urban and non‐urban sites. However, with our current limited knowledge of these interactions and effects, it is difficult to predict how this will affect fitness in urban *versus* non‐urban‐dwelling birds as temperatures warm.

Food quality may also be affected by both urbanisation and temperature in terms of dietary antioxidants including carotenoids. Carotenoids have multiple functions; apart from being antioxidants, they play a role in UV protection and cell membrane stability and are used as colour pigments in the skin, scales and feathers (Britton, 2008). In plants, the synthesisers of carotenoids, heat and UV exposure and air pollution can affect carotenoid function and level (e.g. Camejo et al., 2005; Joshi & Swami, 2009). Effects on carotenoid‐synthesising trees can influence the carotenoid content in the entire food chain, for example, lower carotenoid levels in city trees likely lead to a lower carotenoid content in urban caterpillars and subsequently in urban birds (Isaksson, 2009; Isaksson & Andersson, 2007). This can lead to physiological constraints that affect the morphology of birds, such as carotenoid‐based pigmentation used in mate choice (Olson & Owens, 1998). For example, carotenoid‐based plumage coloration is paler in urban, compared with rural, great tits (Isaksson et al., 2005). The same has been shown for integument colouration of urban Eurasian kestrels (*Falco tinnunculus*; Sumasgutner et al., 2018). As temperatures increase, we can expect a reduction in carotenoid synthesis by primary producers across systems, and these reductions are likely to be exacerbated in urban environments by both air and heat pollution. The impact of this interaction on the relative fitness of urban and non‐urban bird populations is currently unknown.

### 3.3 Microsites

#### 3.3.1 Microsite availability

The use of thermally buffered microsites for daily activities such as foraging is one mechanism by which behaviour may help buffer the effects of elevated air temperatures (Martin et al., 2015). For example, in the Kalahari Desert, several bird species increased their use of trees with the most shade on days >35°C (Martin et al., 2015). In non‐urban areas, shaded microsites include trees, cliffs or banks and topographically variable areas, but shade availability might be limited, particularly in arid environments. However, in urban areas, shaded microsites may be more reliably available, for example in the form of ornamental trees or large areas of shade cast by buildings and other structures, and the contrast between urban and non‐urban areas might be more pronounced in arid regions (see ‘luxury effects’). Such shaded areas could allow birds to continue foraging during hot periods, especially if this shade encompasses resource‐rich areas (e.g. sheltered areas in parks or outdoor cafes that are also rich in anthropogenic food). These differences could theoretically have a profound impact on the heat load experienced by birds in urban *versus* non‐urban areas as temperatures increase. However, currently, we know of no studies which have explored the use of microsites as thermal refugia by urban birds during their daily activity routines (but see Pena et al., 2017 on the positive effect of trees on urban bird diversity).

#### 3.3.2 Microsite quality

Thermal properties of sites used by urban birds for nesting or roosting could differ substantially from these sites in non‐urban areas. For example, the available locations in which to find a nesting or roosting opportunity are often different in urban areas compared with non‐urban areas, which might be especially important for cavity‐nesters relying on tree hollows in natural environments and artificial cavities in urban areas (Mainwaring, 2011). Additionally, the material available to build a nest varies between urban and non‐urban areas (Reynolds et al., 2019). Thus, the thermal conditions of nest and roost sites may differ substantially between urban and non‐urban sites (Mainwaring, 2015; Maziarz et al., 2017; Sudyka et al., 2022) and could alter the responses of birds to climate change, by influencing the heat load they experience in each environment. The impact of elevated temperatures on an individual's fitness may, therefore, vary in direction and magnitude across different sites.

Artificial nest sites in urban areas (e.g. nest boxes, building and roof cavities) are generally less thermally buffered than tree cavities or caves (see microclimate comparisons in Maziarz et al., 2017; Sudyka et al., 2022; and review of nest‐box conductance in Grüebler et al., 2014). The temperatures in such cavities can be several degrees higher than temperatures outside (Maziarz et al., 2017), with consistently higher maximum temperatures, larger temperature amplitudes, and worse insulation from environmental temperatures relative to natural tree cavities (Sudyka et al., 2022). Heat‐related impacts on nest success associated with climate warming might, therefore, be exacerbated in urban, compared with non‐urban, environments. In arid and tropical cities, overheating inside cavities for nesting and roosting will become apparent earlier on, offering a pressing research opportunity to develop mitigating measures, for example, suitable nest box design and improved nest placements (Bideguren et al., 2019) that can then be applied to prevent devastating effects as cities warm. Nest temperature is especially important given that eggs (McCowan & Griffith, 2021; Sharpe et al., 2021) and nestlings (Andreasson et al., 2016; Marques‐Santos & Dingemanse, 2020) are highly sensitive to temperature effects, leaving them potentially more vulnerable to temperature extremes than adults. Recent anecdotal evidence stems from heat‐related mass mortality of swift nestlings in Seville, Spain (Kassam, 2022). Other examples include use of sun‐exposed roofs for nesting by urban seabird colonies (Raven & Coulson, 1997; Soldatini et al., 2008) and higher nestling mortality due to heat stroke or dehydration in thermally unsuitable urban building cavities and planters (discussed for kestrels in Vienna; Sumasgutner et al., 2014), which might presage higher rates of heat‐related breeding failure in urban, compared with non‐urban, areas as temperatures rise.

### 3.4 Air pollution

#### 3.4.1 Air pollution and the redox system

Urban air pollution has important behavioural (e.g. increased preening and homing behaviour), physiological (e.g. increased macrophages in lungs) and morphological effects in birds (reviewed in Sanderfoot & Holloway, 2017). The *redox system* (=oxidation‐reduction status), with its multifaceted and diverse group of antioxidants, plays an important role in reducing these negative effects (e.g. Isaksson, 2010) but is affected by ambient temperatures (e.g. in poultry Miao et al., 2020), potentially making the impact of rising average air temperatures and temperature extremes more severe in urban, compared with non‐urban, areas. In response to increased pro‐oxidative air pollution, up‐regulation of endogenously synthesized antioxidants, such as catalase and superoxide dismutase, is predicted to limit generation of oxidative damage to proteins, lipids and DNA. However, evidence for this effect from urban‐dwelling birds is mixed (e.g. Herrera‐Dueñas et al., 2014; Koivula & Eeva, 2010; Salmón, Stroh, et al., 2018; Salmón, Watson, et al., 2018; Watson et al., 2021). This could be a result of oversimplified predictions regarding how antioxidants or species respond to different levels of urbanisation/air pollution, for example, the antioxidant system might collapse if air pollution is very high (Isaksson, 2020).

In urban areas, as the climate becomes hotter, air pollution worsens, as high air temperatures and increased solar radiation stimulate the production of photochemical smog, as well as ozone, through a combination of meteorological effects, atmospheric chemical reactions and changes to both the rates and types of terrestrial emissions (e.g. Jacob & Winner, 2009; Lee et al., 2006). This will likely aggravate differences between urban and non‐urban areas in terms of air quality. While the negative impact of urban air pollution during periods of high temperatures is well documented in humans (Pourvakhshoori et al., 2020; Stedman, 2004), the thresholds and plasticity of the redox system to combat pollutants at different temperatures have been completely overlooked in wildlife (see also Isaksson, 2020). This means there is a lack of information on taxon‐specific responses to the interaction between high temperature and air pollution, and it is difficult to predict the combined impact of urbanisation and climate warming on birds.

#### 3.4.2 Air pollution and the immune system

Pollutants trigger the immune system and may lead to inflammatory responses, which come with a suite of behavioural and physiological costs in birds (Armour et al., 2020; Bonneaud et al., 2003; Burness et al., 2010; Hegemann et al., 2012, 2018; Hegemann, Matson, Versteegh, et al., 2013; Owen‐Ashley & Wingfield, 2007). Heat stress during development and in adulthood can affect immune function and the ability to mount immune responses, as has been well documented in poultry and domestic mammals (reviewed by Aggarwal & Upadhyay, 2013; Lara & Rostagno, 2013). Interactions of the immune system with other physiological systems (e.g. heat shock proteins, oxidative stress) are also well described in poultry (e.g. Quinteiro‐Filho et al., 2010) although appear not to be well studied in wild birds. Given the results from domestic birds, we predict that heat stress caused by climate change could increase vulnerability to diseases—an effect that might be particularly strong in urban environments, where the UHI effect interacts with air pollution and exposure to pathogens is likely modified (Section 3.1.2). For cold environments, predictions are less clear. On the one hand, release from cold stress could enable individuals to invest more in immune function and develop a better resistance against pathogens. On the other hand, increased temperatures will likely allow for a broader pathogen community.

## 4 CONCLUSIONS AND FUTURE DIRECTIONS

The combined effects of urbanisation and climate change will likely have pronounced consequences for urban birds, and the nature and outcome of interactive effects will differ across different climate types. The availability and quality of resources (e.g. food, water and microsites) and the level of pollution vary between urban and non‐urban areas and across climate types. These factors are likely to mediate the responses of birds to rising temperatures, which are themselves exacerbated in urban areas by the UHI. Interactive effects of urbanisation and rising temperatures are expected to manifest as additive, synergistic and antagonistic across different regions and contexts. Yet, studies directly addressing these effects are almost entirely lacking. A key research question, therefore, is whether the quantity and quality of urban resources, together with pollutant exposure and the UHI, will buffer or exacerbate impacts of warming on the behaviour, physiology and ultimately fitness of urban bird populations, compared with non‐urban conspecifics. This question will need to be addressed across multiple fronts, opening up a broad field of research.

Pioneering studies in temperate regions have so far identified signatures of urbanisation in great tits at the genetic (Salmón et al., 2021) epigenetic (Watson et al., 2021) and transcriptomic (Watson et al., 2017) levels. These data suggest a coordinated and regulated response to the urban environment with changes in expression and methylation of genes involved in stress, immune and inflammatory responses (Watson et al., 2017, 2021). Collectively, the data pinpoint diet and exposure to reactive oxygen species as the likely main drivers of divergence between urban and non‐urban populations, from the epigenetic level through to the phenotypic level. Yet these data are only for one species and a limited number of urban—non‐urban comparisons. Further data are urgently needed to understand whether these results are generalisable across species, taxon groups and regions. For future studies, it would be highly relevant to explore whether the impacts and responses are different for tropical and arid‐dwelling species, especially since we still know so little about genes relevant for coping with high thermal loads in an urban context (but see Park et al., 2019). Furthermore, it remains totally unknown how these genetic, epigenetic and transcriptomic responses to urbanisation will affect the resilience or vulnerability of birds to rising temperatures in terms of behaviour, physiology, fitness and population persistence.

Including thermal behaviour and physiology in future urban wildlife research will, therefore, allow a far better understanding of the resilience of birds to increasing temperatures. Furthermore, future research should address whether buffering effects of urban environments could help species cope with climate warming, or whether the interactive effects between temperature and other urban factors will push birds beyond their thermal tolerance thresholds faster. In urban ecology, the altered conditions and rapid rate of change in cities provide the basis for ‘natural experiments’, with non‐urban environments serving as the reference or control sites. Urban areas can also serve as models for climate change itself (but see Carreiro & Tripler, 2005; Diamond & Martin, 2021; Ziska et al., 2004). The physical environment in cities, which includes higher temperatures, altered hydrological cycles, and elevated CO_2_ concentrations (Kaye et al., 2006), mimics key components of climate change, thus providing opportunities to study responses of biota to such changes. This is particularly important where future predicted climates have no current analogues and hence a species’ ability to cope with these conditions can only be assessed from behavioural thermal thresholds associated with fitness losses (Conradie et al., 2019; Cunningham et al., 2021) and thermal physiological limits (Porter & Kearney, 2009). Taken together, a deeper understanding of the behavioural and physiological mechanisms that mediate species’ responses to the interactive effects of urbanisation and rising temperatures will be needed to unravel the ability of species to adapt to or cope with these challenges.

## AUTHOR CONTRIBUTIONS

The initial spark for this study came from PS and SJC, and the idea and conceptual framework continued to be developed by AH, AA, HW, JFN, MNA and CI. A first manuscript draft was prepared in collaborative writing amongst all co‐authors. The graphs were prepared by PS and SJC. The manuscript was finalised by PS, SJC and CI and approved by all co‐authors.

## FUNDING INFORMATION

This research was funded by the DSI‐NRF Centre of Excellence, a Joint South Africa‐Sweden Research Collaboration (NRF‐STINT grant number STINT160909188048; UID 106777 to AA and PS and SA2016‐6812 to CI and AH) and in part by the Austrian Science Fund (FWF) [Y 1486‐B to PS]. For the purpose of open access, the authors have applied a CC BY public copyright licence to any Author Accepted Manuscript version arising from this submission. PS received furthermore funding from the Claude Leon Foundation; AH was supported by the Swedish Research Council (grant 2018‐04278); and HW was supported by Formas (grant 2016‐00329).

## CONFLICT OF INTEREST STATEMENT

The authors declare no conflict of interest.

## Supporting information


Data S1.

## Data Availability

Data sharing is not applicable to this article as no datasets were generated or analysed during the current study.
