# Supplementary material for: Interactive effects of rising temperatures and urbanisation on birds across different climate zones: A mechanistic perspective
Source: Glob Chang Biol. 2023 Mar 13;29(9):2399–420. doi: 10.1111/gcb.16645 (PMC10947105; doi:10.1111/gcb.16645)
Supplement: Supplementary file 1 — Data S1. [file GCB-29-2399-s001.pdf]

Supporting information and appendices

Interactive effects of rising temperatures and urbanisation on birds across different climatic zones: a mechanistic perspective.

Supplementary Glossary

Air and environmental temperature – Air, environmental and ambient temperatures are terms often used interchangeably in the literature, but in reality they have very distinct meanings. Strictly speaking, air temperature is the temperature of the air itself, normally measured using a temperature probe sheltered within a Stevenson screen at a weather station. The probe is sheltered to remove all influence of radiation, etc, on the temperature reading. Environmental temperature (also known as operative temperature or operative environmental temperature), on the other hand, integrates air temperature, wind, solar and reflected radiation, etc, into a single number approximating the 'thermal challenge' an animal would experience in a particular location in the environment. Environmental temperatures can vary enormously across landscapes under identical air temperature conditions (for example, environmental temperatures in the shade can be tens of degrees °C cooler than environmental temperatures in the sun), and are specific to the animal or object experiencing them as they are influenced by features of the animal itself such as plumage reflectance, etc. 'Ambient temperature' is a term often used loosely in place of either air or environmental temperature, and the exact meaning must often be inferred from context. Some researchers advocate reserving the use of the term ambient temperature to mean controlled temperature conditions in the laboratory (Cunningham et al. 2021).

Anthropogenic global warming – long-term increase in the average temperature of Earth's atmosphere as an effect of the Anthropocene (e.g., human industry and agriculture).

Climate types – following the 5 classifications by Köppen (1931): (1) tropical (or megathermal) climate; (2) arid (or dry) climate; (3) temperate (or mesothermal) climate; (4) continental (or microthermal) climate; (5) polar (or alpine) climate.

Fatty acids (FA) – represent an important energy source for birds and are key structural components of phospholipid bilayers that affect membrane fluidity and cellular function (Hulbert, 2008; Sinensky, 1974). In addition, FAs are crucial for animal development and key modulators of many physiological

processes depending on their chemical nature (Hulbert & Abbott, 2012). The FAs of animals are generally chains of 14 to 22 carbon atoms, which can either be saturated (have no double bonds), monounsaturated (one double bond), or polyunsaturated (several double bonds). The polyunsaturated fatty acids (PUFA) are further classified into e.g., ω-3 or ω-6 PUFAs depending on the position of the most terminal double bond. In birds the ω-3 PUFA α-linolenic acid and the ω-6 PUFA linoleic acid are essential, meaning that they cannot be biosynthesized and therefore must be obtained from the diet. The different classes of FAs have differential effects on physiological processes, and the FA composition of animal tissues can be modulated in response to varying physiological and energetic demands. The FA composition of the diet as well as temperature have individual and combined effects on the FA composition of animals with extended effects on physiology linked to performance, life history, and fitness (Andersson et al., 2018; Ben-Hamo et al., 2011; Guglielmo, 2010; Twining et al., 2016). For instance, basal-, resting-, and peak metabolic rate have all been shown to be affected by the types or identity FAs available for metabolism (Newman et al., 2002; Pierce et al., 2005; Price et al., 2011). In addition, the FA composition of animal tissue affects oxidative stress due to differences in lipid peroxidation susceptibility and effects on membrane fluidity, depending on the degree of FA unsaturation (Hulbert, 2005, 2008). FAs also affect inflammatory responses, with ω-3 PUFAs having anti-inflammatory properties and ω-6 PUFAs pro-inflammatory properties (Hulbert & Abbott, 2012). Expected changes in anthropogenic and natural diet availability, which are likely to be different in urban versus non-urban habitats, in combination with increasing temperatures are therefore likely to affect a large number of physiological traits of birds with unpredictable effects on survival and reproduction.

Immune system – is fundamental for self-maintenance and protects the body from diseases, thereby increasing survival (Roitt et al., 1998). At the same time, it incurs costs in terms of production, maintenance and activation (Hasselquist & Nilsson, 2012; Klasing, 2004). Innate immune function represents the first line of defence, for which activation and maintenance is particularly costly (Klasing, 2004). Innate immune function is related to natural pathogen pressure (Horrocks et al., 2015; Horrocks et al., 2012), and responds to environmental conditions (Hegemann et al., 2012a; Ndithia et al., 2019; Nwaogu et al., 2019). Innate immune function can further be split into baseline (constitutive) immune function and immune responses. Baseline innate immune function represents the ability of an organism to prevent pathogens from entering the body, while an innate immune response is mounted when a pathogen starts replicating and establishing itself in the body. Innate immune responses, in particular acute phase responses, are costly because they include inflammation, fever, reductions of appetite, body mass loss and reduced activity (Adelman et al., 2010; Hart, 1988; Hegemann et al., 2018; Hegemann et al., 2012b; Sköld-Chiriac et al., 2014). Baseline immune function and immune responses

are differently regulated (Hegemann et al., 2012a; Vermeulen et al., 2015; Vinterstare et al., 2019) but reductions in either due to trade-offs with other behavioural and physiological processes can have negative impacts on fitness (Hasselquist & Nilsson, 2012; Hegemann et al., 2013a; Klasing, 2004). Pollutants entering the body will trigger the immune system and may lead to inflammatory responses, which come with a suite of behavioural and physiological costs (Armour et al., 2020; Bonneaud et al., 2003; Burness et al., 2010; Hegemann et al., 2018; Hegemann et al., 2012b; Hegemann et al., 2013b; Owen-Ashley & Wingfield, 2007). Hence, pollution is likely to result in fitness consequences for urban animals as resources are allocated towards immune function and are consequently not available for other physiological and behavioural processes.

Luxury effect – a pattern of higher biodiversity in affluent neighbourhoods, created by the maintenance of green space and the tendency of both humans and other species to favour environmentally desirable areas, while avoiding environmental burdens (Chamberlain et al., 2019). The Luxury Effect is amplified in arid cities and as neighbourhoods age, and is reduced in tropical areas (Chamberlain et al., 2020).

Non-urban – The literature cited uses different habitat types which the authors contrast with “urban” areas. These are either rural areas, mainly consisting of managed agricultural and forestry areas with dispersed human settlements in-between. Or actual natural environments, with little human interference and limited human infrastructure. In this article, we are using the inclusive term “non-urban” that captures both options, so we do not have to explicitly state at every reference used how the original authors contrasted their examples to “urban”. This was done to increase readability.

Redox-system – stands for “oxidation-reduction status” and refers to chemical reactions in which the reactants undergo a change in their oxidation states.

Scholander-Irving model – Metabolic rates (and therefore energy costs) are highest when ambient temperature (i.e. controlled temperature in the laboratory, where most such measurements are done, corresponding most closely to environmental temperature in the field) is low, as endotherms generate extra heat to replace heat lost to the environment (Scholander et al. 1950; Calder and King 1974, see also **Figure 1B**). Energy costs are minimised over a species-specific range of ‘moderate’ ambient temperatures (the “thermoneutral zone”, TNZ). The upper critical limit of the TNZ is defined by a sharp increase in metabolic rate (McNab, 2012) and/or an increase in evaporative water loss (EWL) (IUPS 2001), changes which may or may not co-occur (Mitchell et al., 2018). Water loss rates increase most

rapidly when the thermal gradient for passive heat loss to the environment disappears or is reversed – i.e., when ambient temperature approaches or exceeds the animal's preferred body temperature and evaporative cooling becomes the only effective avenue of heat loss.

Thermal performance curve – are commonly measured in ectothermic animals in the lab (see also Figure 1A) by exposing animals to warmer or cooler ambient temperatures, to which their body temperature then equilibrates. Performance is then measured using righting response, sprint speed, bite strength and other similar measures. Similar performance curves also exist in endothermic animals although these are more difficult to measure empirically, given the physiological control that endotherms exert over their body temperature in the face of changing environmental temperatures.

Urban –The literature cited uses different definitions of "urban", either referring to an urban – non-urban contrast (binary measure) or to a position along an urban – non-urban transition (continuous measure). In our article, we follow the idea of "urban" as a measurable physical structure (e.g., the human footprint in building density or proportion of sealed/unproductive area; known to be associated with human population density, and the exposure to light-, sound- and air-pollution). We refer to areas that are dominated by built structures [> 10 buildings per ha] with more than a minimum density of human residents [typically > 620 km^2 or 10 per ha] and surface cover (> 50%) types (Marzluff, 2001). We are aware that there are others ecologically relevant social gradients linked to urban – non-urban systems, that can be independent of the classical gradients based on urban physical structures. If we, or studies we cite, are referring to such a nuanced view of urbanisation, we go into detail about which measure was used explicitly. See for example the "luxury effect".

Urban heat island effect (UHI) – a measurable increase in urban air and environmental temperatures resulting primarily from the replacement of vegetation with buildings, roads, and other heat-absorbing infrastructure. The heat island effect can result in significant temperature differences between non-urban and urban areas. Impervious surfaces and buildings retain more solar energy than vegetated surfaces during the day, and have lower rates of radiant cooling during the night, resulting in a more pronounced UHI at night (Oke, 1982) that peaks 2-3 h after sunset (Pickett et al., 2001).

Urban heat sink – Cities in arid and semi-arid regions show a modified UHI effect, often exhibiting lower temperatures than non-urban areas during the day (Carnahan & Larson, 1990; Imhoff et al., 2010; Nassar et al., 2016), but with a pronounced heat island effect at night. Urban heat sinks are less documented and more spatially and temporally restricted than UHIs (Zhang et al., 2004).

References

Adelman, J. S., Córdoba-Córdoba, S., Spoelstra, K., Wikelski, M., & Hau, M. (2010). Radiotelemetry reveals variation in fever and sickness behaviours with latitude in a free-living passerine. *Functional Ecology, 24*(4), 813-823. doi:10.1111/j.1365-2435.2010.01702.x

Andersson, M. N., Nilsson, J., Nilsson, J.-Å., & Isaksson, C. (2018). Diet and ambient temperature interact to shape plasma fatty acid composition, basal metabolic rate, and oxidative stress in great tits. *The Journal of Experimental Biology, 221*(24), jeb.186759. doi:10.1242/jeb.186759

Armour, E. M., Bruner, T. L., Hines, J. K., & Butler, M. W. (2020). Low-dose immune challenges result in detectable levels of oxidative damage. *The Journal of Experimental Biology, 223*(6), jeb220095. doi:10.1242/jeb.220095

Ben-Hamo, M., McCue, M. D., McWilliams, S. R., & Pinshow, B. (2011). Dietary fatty acid composition influences tissue lipid profiles and regulation of body temperature in Japanese quail. *Journal of Comparative Physiology B, 181*(6), 807-816. doi:10.1007/s00360-011-0558-2

Bonneaud, C., Mazuc, J., Gonzalez, G., Haussy, C., Chastel, O., Faivre, B., & Sorci, G. (2003). Assessing the Cost of Mounting an Immune Response. *The American Naturalist, 161*(3), 367-379. doi:10.1086/346134

Burness, G., Armstrong, C., Fee, T., & Tilman-Schindel, E. (2010). Is there an energetic-based trade-off between thermoregulation and the acute phase response in zebra finches? *The Journal of Experimental Biology, 213*(8), 1386-1394. doi:10.1242/jeb.027011

Carnahan, W. H., & Larson, R. C. (1990). An analysis of an urban heat sink. *Remote Sensing of Environment, 33*(1), 65-71. doi:10.1016/0034-4257(90)90056-R

Chamberlain, D., Reynolds, C., Amar, A., Henry, D., Caprio, E., & Batáry, P. (2020). Wealth, water and wildlife: Landscape aridity intensifies the urban luxury effect. *Global Ecology and Biogeography, 29*(9), 1595-1605. doi:10.1111/geb.13122

Chamberlain, D. E., Henry, D. A. W., Reynolds, C., Caprio, E., & Amar, A. (2019). The relationship between wealth and biodiversity: A test of the Luxury Effect on bird species richness in the developing world. *Global Change Biology*. doi:10.1111/gcb.14682

Cunningham, S. J., Gardner, J., & Martin, R. O. (2021). Opportunity costs and the response of birds and mammals to climate warming. *Frontiers in Ecology and the Environment, 19*(5), 300-307. doi:10.1002/fee.2324

Guglielmo, C. G. (2010). Move That Fatty Acid: Fuel Selection and Transport in Migratory Birds and Bats. *Integrative and Comparative Biology, 50*(3), 336-345. doi:10.1093/icb/icq097

Hart. (1988). Biological basis of the behavior of sick animals. *Neuroscience & Biobehavioral Reviews, 12*(2), 123-137. doi:10.1016/s0149-7634(88)80004-6

Hasselquist, D., & Nilsson, J.-Å. (2012). Physiological mechanisms mediating costs of immune responses: what can we learn from studies of birds? *Animal Behaviour, 83*(6), 1303-1312. doi:10.1016/j.anbehav.2012.03.025

Hegemann, A., Alcalde Abril, P., Sjöberg, S., Muheim, R., Alerstam, T., Nilsson, J.-Å., & Hasselquist, D. (2018). A mimicked bacterial infection prolongs stopover duration in songbirds—but more pronounced in short- than long-distance migrants. *Journal of Animal Ecology, 87*(6), 1698-1708. doi:10.1111/1365-2656.12895

Hegemann, A., Matson, K., Flinks, H., & Tieleman, B. (2013a). Offspring pay sooner, parents pay later: Experimental manipulation of body mass reveals trade-offs between immune function, reproduction and survival. *Frontiers in Zoology, 10*(1), 77. doi:10.1186/1742-9994-10-77

Hegemann, A., Matson, K. D., Both, C., & Tieleman, B. I. (2012a). Immune function in a free-living bird varies over the annual cycle, but seasonal patterns differ between years. *Oecologia, 170*(3), 605-618. doi:10.1007/s00442-012-2339-3

Hegemann, A., Matson, K. D., Versteegh, M. A., & Tieleman, B. I. (2012b). Wild skylarks seasonally modulate energy budgets but maintain energetically costly inflammatory immune responses throughout the annual cycle. *PLoS ONE, 7*(5), e36358. doi:10.1371/journal.pone.0036358

Hegemann, A., Matson, K. D., Versteegh, M. A., Villegas, A., & Tieleman, B. I. (2013b). Immune response to an endotoxin challenge involves multiple immune parameters and is consistent among the annual-cycle stages of a free-living temperate zone bird. *The Journal of Experimental Biology, 216*(14), 2573-2580.

Horrocks, N. P. C., Hegemann, A., Ostrowski, S., Ndithia, H., Shobrak, M., Williams, J. B., . . . Tieleman, B. I. (2015). Environmental proxies of antigen exposure explain variation in immune investment better than indices of pace of life. *Oecologia, 177*. doi:10.1007/s00442-014-3136-y

Horrocks, N. P. C., Matson, K. D., Shobrak, M., Tinbergen, J. M., & Tieleman, B. I. (2012). Seasonal patterns in immune indices reflect microbial loads on birds but not microbes in the wider environment. *Ecosphere, 3*(2), art19. doi:10.1890/es11-00287.1

Hulbert, A. J. (2005). On the importance of fatty acid composition of membranes for aging. *Journal of Theoretical Biology, 234*(2), 277-288. doi:10.1016/j.jtbi.2004.11.024

Hulbert, A. J. (2008). The links between membrane composition, metabolic rate and lifespan. *Comparative Biochemistry and Physiology Part A: Molecular & Integrative Physiology, 150*(2), 196-203. doi:10.1016/j.cbpa.2006.05.014

Hulbert, A. J., & Abbott, S. K. (2012). Nutritional ecology of essential fatty acids: an evolutionary perspective. *Australian Journal of Zoology, 59*(6), 369-379. doi:10.1071/ZO11064

Imhoff, M. L., Zhang, P., Wolfe, R. E., & Bounoua, L. (2010). Remote sensing of the urban heat island effect across biomes in the continental USA. *Remote Sensing of Environment, 114*(3), 504-513. doi:10.1016/j.rse.2009.10.008

Klasing, K. C. (2004). The costs of immunity. *Dong wu xue bao. [Acta zoologica Sinica], 50*(6), 961-969.

Köppen, W. P. (1931). *Grundriss der Klimakunde*. Berlin: Walter de Gruyter.

Marzluff, J. M. (2001). Worldwide urbanization and its effects on birds. In J. M. Marzluff, R. Bowman, & R. Donnelly (Eds.), *Avian ecology and conservation in an urbanizing world* (pp. 19-48). Boston: Kluwer Academic Publishers.

McNab, B. K. (2012). *Extreme measures: the ecological energetics of birds and mammals*: University of Chicago Press.

Mitchell, D., Snelling, E. P., Hetem, R. S., Maloney, S. K., Strauss, W. M., & Fuller, A. (2018). Revisiting concepts of thermal physiology: Predicting responses of mammals to climate change. *Journal of Animal Ecology, 87*(4), 956-973. doi:10.1111/1365-2656.12818

Nassar, A. K., Blackburn, G. A., & Whyatt, J. D. (2016). Dynamics and controls of urban heat sink and island phenomena in a desert city: Development of a local climate zone scheme using remotely-sensed inputs. *International Journal of Applied Earth Observation and Geoinformation, 51*, 76-90. doi:10.1016/j.jag.2016.05.004

Ndithia, H. K., Versteegh, M. A., Muchai, M., & Tieleman, B. I. (2019). No downregulation of immune function during breeding in two year-round breeding bird species in an equatorial East African environment. *Journal of Avian Biology, 50*(9). doi:10.1111/jav.02151

Newman, R. E., Bryden, W. L., Fleck, E., Ashes, J. R., Buttemer, W. A., Storlien, L. H., & Downing, J. A. (2002). Dietary n-3 and n-6 fatty acids alter avian metabolism: metabolism and abdominal fat deposition. *British Journal of Nutrition, 88*(1), 11-18. doi:10.1079/BJN2002580

Nwaogu, C. J., Cresswell, W., Versteegh, M. A., & Tieleman, B. I. (2019). Seasonal differences in baseline innate immune function are better explained by environment than annual cycle stage in a year-round breeding tropical songbird. *Journal of Animal Ecology, 88*(4), 537-553. doi:10.1111/1365-2656.12948

Oke, T. R. (1982). The energetic basis of the urban heat island. *Quarterly Journal of the Royal Meteorological Society, 108*(455), 1-24. doi:10.1002/qj.49710845502

Owen-Ashley, N. T., & Wingfield, J. C. (2007). Acute phase responses of passerine birds: characterization and seasonal variation. *Journal of Ornithology, 148*(2), 583-591. doi:10.1007/s10336-007-0197-2

Pickett, S. T., Cadenasso, M. L., Grove, J. M., Nilon, C. H., Pouyat, R. V., Zipperer, W. C., & Costanza, R. (2001). Urban ecological systems: linking terrestrial ecological, physical, and socioeconomic components of metropolitan areas. *Annual Review of Ecology and Systematics, 32*(1), 127-157.

Pierce, B. J., McWilliams, S. R., O'Connor, T. P., Place, A. R., & Guglielmo, C. G. (2005). Effect of dietary fatty acid composition on depot fat and exercise performance in a migrating songbird, the red-eyed vireo. *The Journal of Experimental Biology, 208*(7), 1277-1285. doi:10.1242/jeb.01493

Price, E. R., Staples, J. F., Milligan, C. L., & Guglielmo, C. G. (2011). Carnitine palmitoyl transferase activity and whole muscle oxidation rates vary with fatty acid substrate in avian flight muscles. *Journal of Comparative Physiology B, 181*(4), 565-573. doi:10.1007/s00360-010-0542-2

Roitt, I., Brostoff, J., & Male, D. (1998). *Immunology* (5 ed.). London, Mosby.

Sinensky, M. (1974). Homeoviscous Adaptation—A Homeostatic Process that Regulates the Viscosity of Membrane Lipids in Escherichia coli. *Proceedings of the National Academy of Sciences, 71*(2), 522-525. doi:10.1073/pnas.71.2.522

Sköld-Chiriac, S., Nord, A., Nilsson, J., & Hasselquist, D. (2014). Physiological and behavioral responses to an acute-phase response in zebra finches: immediate and short-term effects. *Physiological and Biochemical Zoology, 87*. doi:10.1086/674789

Twining, C. W., Brenna, J. T., Lawrence, P., Shipley, J. R., Tollefson, T. N., & Winkler, D. W. (2016). Omega-3 long-chain polyunsaturated fatty acids support aerial insectivore performance more than food quantity. *Proceedings of the National Academy of Sciences, 113*(39), 10920-10925. doi:10.1073/pnas.1603998113

Vermeulen, A., Muller, W., Matson, K. D., Tieleman, B. I., Bervoets, L., & Eens, M. (2015). Sources of variation in innate immunity in great tit nestlings living along a metal pollution gradient: an individual-based approach. *Science of the Total Environment, 508*. doi:10.1016/j.scitotenv.2014.11.095

Vinterstare, J., Hegemann, A., Nilsson, P. A., Hulthén, K., & Brönmark, C. (2019). Defence versus defence: Are crucian carp trading off immune function against predator-induced morphology? *Journal of Animal Ecology, 88*(10), 1510-1521. doi:10.1111/1365-2656.13047

Watson, H., Videvall, E., Andersson, M. N., & Isaksson, C. (2017). Transcriptome analysis of a wild bird reveals physiological responses to the urban environment. *Scientific Reports, 7*, 44180. doi:10.1038/srep44180

Zhang, X., Friedl, M. A., Schaaf, C. B., Strahler, A. H., & Schneider, A. (2004). The footprint of urban climates on vegetation phenology. *Geophysical Research Letters, 31*(12), L12209. doi:10.1029/2004GL020137
